# Avian Alarm Calls Do Not Induce Anti-Predator Response in Three Anuran Species

**DOI:** 10.3390/ani12243537

**Published:** 2022-12-14

**Authors:** Longhui Zhao, Yuanyu Qin, Jichao Wang, Wei Liang

**Affiliations:** Ministry of Education Key Laboratory for Ecology of Tropical Islands, Key Laboratory of Tropical Animal and Plant Ecology of Hainan Province, College of Life Sciences, Hainan Normal University, Haikou 571158, China

**Keywords:** anti-predator behavior, anuran, heterospecific eavesdropping, sound playback

## Abstract

**Simple Summary:**

Many birds and mammals are capable of eavesdropping on heterospecific alarm calls, yet it remains unclear whether other taxa (e.g., amphibians) can exploit such acoustic signals. Here, we tested whether three anuran species showed anti-predator response (e.g., escaping behavior) to territory song and different alarm calls of Japanese Tits (*Parus minor*). We found that all species performed no response to the territory song and alarm calls. In spite of negative results, this study provides valuable information on the cognitive processes of amphibian species.

**Abstract:**

Many species produce alarm calls in response to predators, and the anti-predator signals are often used by other species. Eavesdropping on heterospecific alarm calls has been widely found in bird and mammal species. Other taxa, such as reptiles and amphibians, however, receive limited attention at present. Here, we selected three types of alarm calls of Japanese Tits (*Parus minor*) that were evoked by the Siberian Chipmunk (*Eutamias sibiricus*), Eurasian Sparrow Hawk (*Accipiter nisus*), and model snake (*Elaphe* spp.), respectively, and then carried out playback experiments to test whether three frog species changed their behaviors in response to the three treatments of Japanese Tit calls while the tit’s territory song was used as a control. The results showed that Little Torrent Frogs (*Amolops torrentis*), Ornamented Pygmy Frogs (*Microhyla fissipes*) and Spot-legged Treefrogs (*Polypedates megacephalus*) did not jump off their positions in response to the same four acoustic signals. They also did not change their calling behaviors in response to the alarm calls of Japanese Tits. This study found no evidence that these anuran species can eavesdrop on heterospecific tits’ alarm signals.

## 1. Introduction

Animals often obtain information about their habitats from diverse sources, to increase certainty and make adaptive decisions [1,2,3]. Acoustic signals are one of the most important information carriers, and are widely used in a variety of activities such as social interactions [4]. Many species, such as birds and mammals, emit specific calls (e.g., alarm calls) in response to the presence or attack of predators [5,6]. Alarm calls can advertise the type of predators. For instance, some parental birds can use acoustically distinctive signals to encode the information of different nest predators [7,8], although there is variation in note types of alarm calls among geographic populations [9], while nestlings vary their predator-avoidance behaviors when presented with different alarm calls from parents [10]. Alarm calls can also advertise the urgency or degree of threat [6,11,12]. For example, the Australian magpie (*Cracticus tibicen*) gives a higher frequency of alarm calls in risky situations [13], while in some terrestrial rodents, individuals can change the type of their acoustic signals when exposed to different degrees of risky situations [14]. In animal communities, alarm calls provide important public information and more opportunity of avoiding predators for those concomitant species.

Alarm calls not only provide complex information for conspecific communication, but also have ecological importance in interspecific interactions [3]. Sound playbacks on birds, for instance, suggest that eavesdropping on the alarm calls of other species leads to a reduction in vigilance behaviors and an increase in foraging success in some species [15,16,17], while some other species show more vigilance behaviors when they hear heterospecific alarm calls (e.g., [18,19,20]). Research on several mammals shows that squirrels and marmots quickly flee to refuge after the playback of other species’ alarm calls [21,22]. In addition, some animals can learn to recognize other species’ alarm calls, which may lead to the spread of predator or risk recognition and the evolution of eavesdropping diversity [23,24]. Apart from these direct and indirect benefits, as compared with conspecifics, the exploitation of heterospecific alarm calls could also give animals more available information about dangers and reduce their cost of detecting risk [3]. Although such cross-species eavesdropping has been reported in many taxa, most research is mainly conducted in bird and mammal species. This anti-predator defense strategy is less studied in other animal groups such as amphibians and reptiles.

Anurans (frogs and toads) primarily communicate with acoustic signals [25]. In the wild, their predators (e.g., birds) often produce calls and use acoustic signals to transmit diverse conspecific or heterogenous information. It would be adaptive if anurans utilize the calls of other sympatric species. Surprisingly, few studies have explored whether they can eavesdrop on heterospecific calls [26]. Tits are a widely distributed species and can generate alarm calls when exposed to potential predators. Japanese Tits (*Parus minor*), for instance, often emit different calls in response to the presence of snakes and other bird predators [8,27]. Here, we used different acoustic signals of Japanese Tits to investigate whether avian alarm calls induce anti-predator responses in anuran species.

In this study, we examined the behavioral responses of three frog species to a territory song (control group) and three alarm calls of Japanese Tits. The three alarm calls were evoked by snake, squirrel, and hawk predators, respectively. We used sound playback experiments to test whether calling Little Torrent Frogs (*Amolops torrentis*) escaped or changed calling behaviors in response to the alarm calls of Japanese Tits. Acoustic signals can expose animals’ positions. Calling males thus may face greater risks than silent males. Therefore, we further compared the behavioral differences between calling males and silent males in different sound playback tests of the torrent frog. Differences in habitats may bring different prey pressures to anuran species. Apart from the torrent frog species, we also conducted the same experiments in a terrestrial frog species, that is, Ornamented Pygmy Frog (*Microhyla fissipes*), and a dendrocola frog species, that is, the Spot-legged Treefrog (*Polypedates megacephalus*). In the two species, we examined whether males jumped or ceased calling in response to three treatments of alarm calls.

## 2. Materials and Methods

### 2.1. Study Site

Little Torrent Frogs and Ornamented Pygmy Frogs were tested in Wuzhishan Natural Reserve (109°32′−43′ E, 18°48′−59′ N), Hainan Province, China. The study site of Little Torrent Frogs was a stretch of mountain stream (~300 m) around Shuiman Station, while the site of Ornamented Pygmy Frogs was a field around Shuiman Village. During sound playbacks, 3–5 males were often close and calling to focal individuals at high density chorus. Average annual air temperature in this site was about 22.4 °C [28]. The Little Torrent Frog and Ornamented Pygmy Frog are dominant species in the Wuzhishan Natural Reserve. Spot-legged Treefrogs were tested in Dongzhaigang Natural Reserve (110°32′−37′ E, 19°51′−20°1′ N), Hainan Province, China, with average annual air temperature being about 23.8 °C [29]. The study site was a bush forest close to Honglin Road. The Spot-legged Treefrog is a dominant species in the Dongzhaigang Natural Reserve. For this species, 2–3 males were often close and calling to focal individuals at high density chorus.

### 2.2. Sound Designs

**Figure 1:**
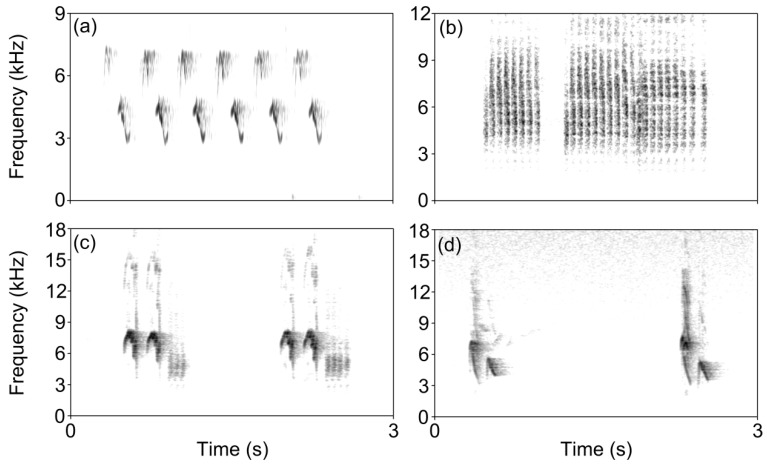
Spectrograms of four groups of Japanese Tit calls. (**a**) territory song; (**b**) alarm call evoked by the Siberian Chipmunk; (**c**) alarm call evoked by the Sparrow Hawk; (**d**) alarm call evoked by the snake.

Four groups of Japanese Tit calls were used in present study (Figure 1). One type was territory song of this species with the absence of predators, while the other three types were predator-evoked calling that was produced in response to the presence of Siberian Chipmunk (*Eutamias sibiricus*), Eurasian Sparrow Hawk (*Accipiter nisus*) and model snake (*Elaphe* spp.), respectively. For each group, we selected two typical recordings with the length of 1–2 min (see [8,27]). We adjusted all sound files to a similar intensity and lengthened each of them to 3 min. Our study had showed that Little Torrent Frogs, Ornamented Pygmy Frogs, and Spot-legged Treefrogs did not escape and cease calling in response to white noise playbacks (Table 1; Longhui Zhao et al., unpublished data). In the present study, we treated the territory song of Japanese Tits as a further control.

### 2.3. Sound Playback Experiments

Playback experiments were conducted in May–June 2022. After male Little Torrent Frogs were located, we presented four types of call stimuli stochastically using a small speaker (Clip3, JBL, Northridge, CA, USA) from a distance of ~1 m. Sound pressure levels were about 80 dB at such distance, which is approximate to the call intensity of this species in natural environments [30]. The interval between different call stimuli was set as 2 min, in order to avoid the potential effect of one test on the other. Animal responses to different call playbacks were recorded using a digital video camera (FDR-AX40, Sony, Tokyo, Japan). Little Torrent Frogs are nycterohemeral species, but they were only tested in warm sunshine periods (i.e., daily 8:00 a.m.–14:00 p.m.) in order to assure the activity of the frog was synchronous with Japanese tits as well as its predators and make the light condition optimal for animal visual communication. In a previous survey, male Little Torrent Frogs were observed to have a specific territory and often stay at a fixed position for some days [28]. Therefore, we selected males from different locations to conduct sound playbacks in this study. Moreover, frogs in extreme high-intensity noise were not used due to the potential severe masking on call stimuli. Both silent frogs and calling frogs were tested in the present study.

Ornamented Pygmy Frogs and Spot-legged Treefrogs were tested at night. Their playback procedures were the same as with Little Torrent Frogs. The playback distance, however, was longer than the Little Torrent Frogs (~1.5 m). In such distance, four sound stimuli were about 78 dB during playback tests. Their behavioral performances were determined by calling and locomotor sounds. During sound playbacks, we firstly judged whether calling frogs stayed at their original positions according to their vocal behaviors. If one ceased calling, we then checked whether it had escaped or left, dependent on weak light. In the two species, almost all individuals remained calling during sound playbacks.

### 2.4. Data Analyses

We depended on video and sound recordings to determine whether Little Torrent Frogs, Ornamented Pygmy Frogs and Spot-legged Treefrogs escaped in response to the territory song of Japanese Tits and their alarm calls evoked by three predators. We used binominal tests (two-tailed) to examine whether animals varied their escaping proportion in response to different call playbacks. The hypothesized probability for this test was 0.5. We employed Fisher’s exact tests (two-tailed) to evaluate whether four groups showed differences in the probability of escaping and compared behavioral differences between silent torrent frogs and calling torrent frogs. All statistical analyses were performed in R 4.1.0 [31]. *p* < 0.05 was used as a significance level in all tests.

## 3. Results

### 3.1. Responses of Little Torrent Frogs to Sound Playbacks

A total of 31 male Little Torrent Frogs (18 silent males and 13 calling frogs) were tested in playback experiments. The proportion of not-jumped males was significantly higher than jumped males when broadcast with one territory and three predator-evoked calls of Japanese Tits (binomial test: *p* < 0.001 in all experiments; Table 2). Four groups had no difference in the probability of escaping (Fisher’s exact test: *p* = 1). Moreover, silent males and calling males did not differ in their behaviors when exposed to territory songs (silent males vs. calling males: 18/0 vs. 12/1, *p* = 0.419), squirrel-evoked alarm calls (silent males vs. calling males: 18/0 vs. 13/0, *p* = 1), snake-evoked alarm calls (silent males vs. calling males: 18/0 vs. 13/0, *p* = 1), and hawk-evoked alarm calls (silent males vs. calling males: 18/0 vs. 13/0, *p* =1). Among all calling individuals, only one sample was observed to stop calling in response to territory song and hawk-evoked alarm call, while no sample became silent when presented with squirrel-evoked alarm call and snake-evoked alarm call. Thus, both silent males and calling males showed no response to the playbacks of territory and predator-induced calls of Japanese Tits.

### 3.2. Responses of Ornamented Pygmy Frogs and Spot-Legged Treefrogs to Sound Playbacks

In total, 21 male Ornamented Pygmy Frogs and 24 male Spot-legged Treefrogs were tested, respectively, in playback experiments. The Ornamented Pygmy Frogs did not jump off their positions in response to the presence of one territory and three predator-evoked calls of Japanese Tits (binomial test: *p* < 0.001 in all experiments; Table 2). In this species, four groups showed no difference in the probability of escaping (Fisher’s exact test: *p* = 1). The Spot-legged Treefrogs also did not escape when presented with different Japanese Tit calls (binomial test: *p* < 0.001 in all experiments; Table 2). Again, there was no difference in escaping behavior between different groups (Fisher’s exact test: *p* = 1). In two species, none of the not-escaped samples were observed to cease calling in response to four tested groups. Therefore, neither frog species had a response to the playbacks of territory and predator-induced calls of Japanese Tits.

## 4. Discussion

We found that Little Torrent Frogs, Ornamented Pygmy Frogs, and Spot-legged Treefrogs did not jump off their positions when exposed to one territory and three predator-evoked calls of Japanese Tits. They also did not cease calling in response to four types of calls. Furthermore, there were no differences between calling torrent frogs and silent torrent frogs, although calling individuals may have greater pressures from predators. Consequently, avian alarm calls do not evoke anti-predator responses in anuran species in this study.

Research on several frogs and lizards have demonstrated that amphibians and reptiles may have the ability to use heterospecific alarm calls [26,32,33,34]. In the Hainan Tropical Rainforest, we have observed that snakes, such as *Sinonatrix percarinata*, often prey on Ornamented Pygmy Frogs, Spot-legged Treefrogs, and some other anurans in the wild. Meanwhile, birds are also the potential predators of anuran species. Moreover, tits are native species to Hainan Island and share common areas with the Little Torrent Frog, Ornamented Pygmy Frog, and Spot-legged Treefrog [35]. Thus, it would be adaptive if these species could exploit tits’ alarm calls that warn of snakes, birds and other potential predators. Our results, however, did not show that Little Torrent Frogs (both calling males and silent males) can use heterospecific alarm signals evoked by snake, bird, and squirrel predators of Japanese Tits. Our results also showed that Ornamented Pygmy Frogs and Spot-legged Treefrogs performed no anti-predator responses to different tit alarm calls. This study failed to provide evidence for anuran heterospecific eavesdropping on avian alarm calls.

Stream habitats are often characterized by fast flowing water which can generate continuous background noise [36,37]. Little Torrent Frogs prefer to live in noisy positions characterized by lots of stones [30]. In natural environments, Japanese Tit calls may be masked by stream noise and not easily detected by torrent frogs. These factors may prevent a strong acoustic association between torrent frogs and sympatric Japanese Tits, as well as other species. The investigations on two frog species that have different life history, however, also showed similar results with the Little Torrent Frog. Unlike most anuran species, Little Torrent Frogs have high activity during the day. This species shares similar colors with the habitat background, but differs distinctly in their white vocal sacs [38]. The similarity between body color and background color is beneficial to avoid predators [39,40,41,42]. Visual camouflage can be very effective by daylight. It is possible that visual information has a more important role than acoustic signals in the anti-predator strategy of this species.

In this study, three treatments of tit alarm calls include the information of snake, bird, and squirrel predators, respectively. The bird and squirrel predators of tits may have a low probability of predation on Little Torrent Frogs, Ornamented Pygmy Frogs, and Spot-legged Treefrogs, which is also a possible reason of negative results. Therefore, future research needs to further examine those avian alarm calls evoked by predators that have closer prey–predator relationships with anuran species. Due to the high-density chorus and difficulties in qualifying the number of calls in some species, this study only observed whether frogs ceased calling in response to playbacks and did not assess their calling activity exactly (e.g., call rate in different playback periods) as well as other behaviors (e.g., flight initiation distance). Indoor control tests or further investigations on low-density chorus and other behaviors are needed in future.

## 5. Conclusions

Taken together, our results suggest that three frog species do not eavesdrop on the alarm calls of Japanese Tits evoked by different types of predators. Visual signals may provide more anti-predator information for these species, which needs further research in the future. In spite of our negative results, the lack of eavesdropping on avian alarm signals in the three frog species provides valuable information on the cognitive decision-making processes of amphibian species.

## Figures and Tables

**Table 1:** Responses of three frog species to white noise playbacks.

Species	Sample Size	Number Escaped/Not Escaped	Number Silent/Not Silent
Little Torrent Frog	13	1/12	0/13
Ornamented Pygmy Frog	15	0/15	0/15
Spot-legged Treefrog	13	0/13	0/13

**Table 2:** Movement responses of Little Torrent Frogs, Ornamented Pygmy Frogs and Spot-legged Treefrogs to different calls.

Species	Call Type	Number Not Escaped	Number Escaped	*p*
Little Torrent Frog	TS	30	1	<0.001
	SCEC	31	0	<0.001
	SEC	31	0	<0.001
	SHEC	31	0	<0.001
Ornamented Pygmy Frog	TS	20	1	<0.001
	SCEC	21	0	<0.001
	SEC	21	0	<0.001
	SHEC	21	0	<0.001
Spot-legged Treefrog	TS	24	0	<0.001
	SCEC	23	0	<0.001
	SEC	23	1	<0.001
	SHEC	23	1	<0.001

Note: TS, SCEC, SEC, and SHEC represent territory song, Siberian Chipmunk evoked call, snake evoked call, and Sparrow Hawk evoked call, respectively.

## Data Availability

Not applicable.
